# Redefining Obesity in the Indonesian Population: The Critical Role of Waist-to-Height Ratio in Screening for Diabetes Mellitus and Hypertension

**DOI:** 10.1155/jnme/5815261

**Published:** 2025-09-02

**Authors:** Ratu Ayu Dewi Sartika, Fathimah Sulistyowati Sigit, Nurul Husna Mohd Shukri, Edy Purwanto, Jasrida Yunita, Pika Novriani Lubis

**Affiliations:** ^1^Faculty of Public Health, Universitas Indonesia, Depok, Indonesia; ^2^Metabolic Disorder, Cardiovascular, and Aging Research Cluster, Faculty of Medicine, Indonesia Medical Education and Research Institute, Universitas Indonesia, Jakarta, Indonesia; ^3^Jabatan Pemakanan dan Dietetik, Universiti Putra Malaysia, Serdang, Selangor, Malaysia; ^4^Department of Economics, Faculty of Economics and Business, Airlangga University, Surabaya, Indonesia; ^5^SurveyMETER, Yogyakarta, Indonesia; ^6^Sekolah Tinggi Ilmu Kesehatan Hang Tuah, Pekanbaru, Indonesia

**Keywords:** body mass index (BMI), diabetes mellitus (DM), hypertension (HTN), waist circumference (WC), waist-to-height ratio (WHtR), waist-to-hip ratio (WHR)

## Abstract

**Objectives:** Waist-to-height ratio (WHtR) is an alternative index to evaluate metabolic health and predict the risk of estimating the impact of adiposity on cardiometabolic diseases. Despite the significance, the diagnostic performance of WHtR has not been extensively investigated in large epidemiological studies in Indonesia. Therefore, this study aimed to investigate anthropometric indexes (body mass index [BMI], waist circumference [WC], waist-to-hip ratio [WHR], and WHtR) with the best clinically accurate and diagnostic performance in detecting the prevalence of diabetes mellitus (DM) and hypertension (HTN) in the Indonesian population.

**Methods:** This study used a cross-sectional method to analyze big data of 7699 individuals from the Indonesian Family Life Survey. The diagnostic performance of each anthropometric index was analyzed using the receiver operating characteristics (ROC) curve model in the SPSS and MedCalc applications. Furthermore, the associations of anthropometric indexes with DM and HTN were evaluated using logistic regression adjusted for sociodemographic confounders.

**Results:** WHtR showed the highest area under the curve (AUC) for detecting DM in men (0.731 [0.679–0.784]), as well as HTN in both men (0.650 [0.629–0.671]) and women (0.615 [0.598–0.633]). Although often negligible, the discrepancies had overlapping 95% confidence intervals with other indexes. WHtR also showed the strongest association with both DM (AOR [95% CI]: 3.166 [2.416–4.150]) and HTN (1.938 [1.703–2.206]). Lower cutoffs for BMI (22.72 kg/m^2^) and WC (83.35 cm) enhanced sensitivity for DM and HTN detection, particularly in men.

**Discussion:** WHtR outperformed BMI, WC, and WHR in detecting DM and HTN in the Indonesian population. Additionally, lower cutoffs for overall (BMI) and abdominal obesity (WC) should be considered to enhance the sensitivity of anthropometric indexes in screening for cardiometabolic diseases in the population.

## 1. Introduction

Metabolic syndrome (MetS) is composed of several diseases, such as dysregulated lipid metabolism, abdominal obesity, dysglycemia, and hypertension (HTN). These diseases significantly increase the risk of diabetes mellitus (DM) and cardiovascular disease (CVD). As a global epidemic, MetS primarily affects underdeveloped nations [1, 2]. In the South–East Asia Region (SEAR), the prevalence of MetS varies between 15% and 29% [3]. MetS-related outcomes, including DM and CVD, consistently pose a substantial national burden in Indonesia, ranking among the top three causes of premature mortality and disability-adjusted life years [4].

Many anthropometric indexes have been used to evaluate and estimate the effect of obesity on disease cardiometabolic risks, particularly DM and CVD [5]. A simple and practical anthropometric measure that is commonly used is body mass index (BMI), which can be an indicator to estimate relative adiposity without providing information regarding body composition. BMI may offer restricted insight into metabolic health and introduce bias in body fat estimation due to the inability to distinguish between fat and fat-free (muscle) mass [6]. For instance, athletes with muscular builds may be categorized as overweight according to BMI criteria despite having a low amount of body fat. To overcome these limitations, alternative measures that consider fat deposition in the abdominal region, such as waist circumference (WC), waist-to-hip ratio (WHR), and waist-to-height ratio (WHtR), are used in predicting cardiometabolic disease risks [7, 8]. This is because abdominal obesity fat distribution showing visceral fat accumulation is associated with metabolic dysregulation, such as lowered glucose tolerance, reduced insulin sensitivity, and unfavorable lipid profile, which are risk factors for DM and CVD [9].

Previous studies had differing results on anthropometric indexes, with the highest accuracies for estimating cardiometabolic risk from obesity. For example, a meta-analysis showed that WC was superior to BMI, WHR, and WHtR for detecting cardiovascular risk, although the evaluation only focused on correlation coefficients [10]. Other studies identified WHR as the anthropometric index that was most strongly associated with the risk of DM, while WHtR was used for HTN [11, 12]. Another report showed that WHtR was more strongly associated with the incidence of DM compared to BMI and WHR [13]. Therefore, identifying the index with the best performance in predicting cardiometabolic risks is essential to developing effective prevention programs for chronic noncommunicable diseases.

Currently, many nation-specific studies have been conducted to determine the cutoffs for anthropometric indexes in different populations. This is because the general, international anthropometric cutoffs can be inaccurate for predicting obesity-related cardiometabolic risks in various ethnic populations [14]. Related studies on the matter in Indonesia are also still rare, becoming a knowledge gap to be filled. Therefore, this nationally representative study with a large sample was performed to identify anthropometric indexes that had the best diagnostic performance in assessing the risks of DM and HTN. Analysis was also carried out to determine the accuracy of the current anthropometric cutoffs detecting cardiometabolic risks, comparing the diagnostic values of the current standard with the new, proposed cutoffs from the model developed. The results were expected to provide valuable insights into the significance of these indexes in enhancing early diagnosis of metabolic disorders.

## 2. Materials and Methods

**Figure 1:**
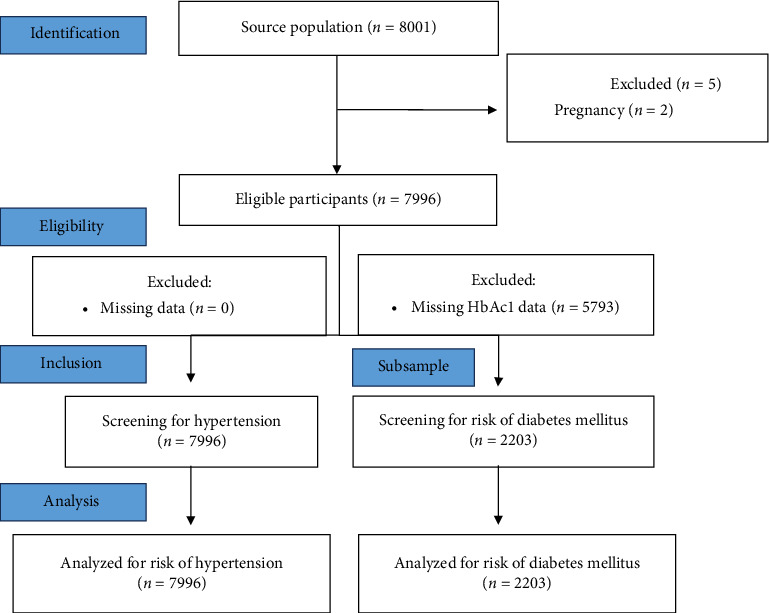
STROBE flow diagram of cross-sectional studies.

This study analyzed the secondary data from the Indonesian Family Life Survey (IFLS). According to the definition, IFLS is a prospective cohort survey with a large sample size, representing approximately 83% of the diversity of ethnicity, geographical distribution, and sociodemographic characteristics of the total Indonesian population. With repeated measurements every 7 years, the survey provides robust insights. In this observational study, the cross-sectional data were obtained from the 2014 survey. The sample was randomly selected from 312 enumeration areas across 13 provinces. This included over-sampling of urban areas in smaller provinces to facilitate urban-rural comparisons, leading to a total of 7224 households. More details about the selection sample can be assessed on the IFLS website [15]. In the analysis, only those who were nonpregnant adults aged ≥ 18 years without pre-existing DM and HTN at baseline were included. A total of 7699 adults represented the number of participants, indicating the absence of exclusion data. All samples at baseline participated in the survey, completing the general questionnaires and undergoing comprehensive anthropometric and blood pressure measurements. However, for investigating DM in the population, the subsample was selected (*n* = 2203) due to technical obstacles or participants' rejections. The capillary blood HbA1c test was administered exclusively to those subsamples. Comprehensive explanations can be found at https://sites.rand.org/labor/family/software_and_data/FLS/IFLS/IFLS2/doc/volume2.zip). The Strengthening and Reporting of Observational Studies in Epidemiology (STROBE) flow diagram showed the sample selection method (Figure 1).

### 2.1. Exposure Variables: Anthropometric Indexes

The exposure variables in this study are anthropometric measures of individuals, which are BMI, WC, WHR, and WHtR, while the outcome variables are HbA1c level and systolic and diastolic blood pressures. BMI was calculated as body weight in kilograms divided by the square of height in meters (kg/m^2^). Body weight was measured with a digital scale to the nearest 0.1 kg. Height was measured without shoes using a portable stadiometer to the nearest 0.1 cm. Furthermore, WC was measured with a flexible tape halfway between the iliac crest and the lowest rib. To define obesity based on each index, the Asian population- and sex-specific cutoffs were used, which were > 25 kg/m^2^ for BMI [16], > 90 cm in men and > 80 cm in women for WC [17], > 0.9 in men and > 0.85 in women for WHR [18], and > 0.5 for WHtR [19].

### 2.2. Outcome Variables: HbA1c and Blood Pressures

HbA1c levels of individuals who were randomly selected for blood tests (*n* = 2203) were used to define the risk of DM. In this study, HbA1c sample was obtained with dried blood spot (DBS) method, where capillary blood was dripped or blotted onto a specific filter paper and allowed to dry before analysis. This method was selected for logistical factors, due to difficulties in obtaining and transferring blood samples from rural or remote areas to laboratories with adequate standard clinical chemistry examination facilities. Based on American Diabetes Association (ADA) and World Health Organization (WHO), HbA1c level can be used as a diagnostic criterion for DM, with 6.5% set for the cutoff to diagnose DM [20]. Blood pressure was assessed in the total population using a digital sphygmomanometer on the left arm after 5 minutes of rest and the average of three measurements was used in the analysis. HTN is defined as a systolic blood pressure of ≥ 140 mmHg and a diastolic blood pressure of ≥ 90 mmHg [21].

### 2.3. Statistical Analyses

Population characteristics were described as mean and standard deviation (SD) for continuous variables and proportion (%) for categorical variables. To investigate the associations of anthropometric indexes with DM and HTN, bivariate and multivariable logistic regressions were performed with BMI, WC, WHR, and WHtR as the exposure, while the prevalence of DM and HTN served as the outcomes. Variables with a *p*-value < 0.25 in bivariate analysis were considered potential confounders and included in the final model in multivariate analysis. The association was analyzed with adjusted cofounder variables such as sociodemographic and lifestyle factors. These included age, sex (men/women), marital status (married/unmarried), level of education (high school or higher/junior high school or lower), working status (working/not working), living area (urban/rural), depression (yes/no), quality of sleep (poor/good), physical activity (active/inactive), smoking status (smoking/not smoking), and dietary factors such as staple food, protein, sugary foods, fruits, and vegetable consumption (days/week), which were obtained from the survey questionnaire. The results were presented as odds ratios (ORs) with 95% confidence intervals (CIs) to describe the associations between the exposures and the outcomes.

To evaluate the diagnostic performance of each anthropometric index in detecting DM and HTN, the Medcalc application (v.19.4.1) was used to obtain positive predictive value (PPV), negative predictive value (NPV), sensitivity, and specificity, which determined the magnitude of diagnostic accuracy [22]. Additionally, ROC analysis was performed using IBM SPSS Statistics Version 23.0 software (IBM Co., Armonk, NY, USA, RRID: SCR_002865) to generate area under the curve (AUC), a feature absent in Medcalc. The ROC method evaluates how well a diagnostic test can discriminate the presence of a disease by examining the width of the AUC, which presents the trade-off between sensitivity and specificity. For each anthropometric index, the optimal cutoff point was identified and proposed using the Youden index, which maximized the sum of sensitivity and specificity.

AUC ranges from 0 to 1, with a larger value reflecting a better diagnostic performance of a test in detecting the presence of a target disease. A test is considered to have acceptable performance with an AUC of ≥ 0.6 [23]. To identify how strongly the exposure and outcome variables were correlated, the Spearman correlation coefficients were tested between all anthropometric indexes and the cardiometabolic markers (HbA1c, systolic, and diastolic blood pressures). This study adhered to STROBE guidelines for reporting.

## 3. Results

### 3.1. Descriptive Characteristics of the Survey Population

The results showed that there were more women (*n* = 4507) than men (*n* = 3192) in this study. The mean (SD) age is 52.59 (11.22) years. The mean (SD) of anthropometric indexes of the population, as stratified by sex, were described in Table 1. Women generally had larger waist and hip circumferences but were shorter than men. Women also had a higher mean (SD) BMI than men (women: 24.8 [4.7] kg/m^2^; men: 22.6 [3.8] kg/m^2^), showing an average BMI that was above the normal cutoff and in the range of overweight.

From the total study population (*n* = 7669), there were 3219 cases of HTN (41.8%). Among the subsample of participants who passed through capillary blood tests (*n* = 2203), 290 individuals had DM (13.2%). Sex distribution was similar in the prevalence of DM (women: 13.0%; men: 13.4%), but slightly higher in women (44.3%) than men (38.3%) for HTN (Table 1).

Table 2 shows the characteristics of the population as stratified by disease status. Individuals with HTN or DM were generally older than those without the conditions. Additionally, individuals with diseases were more likely to have both overall and abdominal obesity, due to higher levels of all anthropometric indexes. DM and HTN often coexisted, as average HbA1c levels were relatively higher in HTN, and blood pressure was elevated in those with DM.

### 3.2. The Associations of Anthropometric Indexes With DM and HTN

Table 3 shows the associations of BMI, WC, WHR, and WHtR with DM and HTN. Compared to other anthropometric indexes, WHtR had the strongest associations with both diseases after controlling confounders, including sociodemographic variables, smoking status, and physical activity (adjusted OR [95% CI]: 3.17 [2.42–4.15] for DM and 2.13 [1.93–2.36] for HTN). Other variables were excluded from the model due to the lack of significance in the bivariate association (Table 2). These associations remained stable even after further adjustment for BMI (3.17 [2.42–4.15] for DM; 1.94 [1.70–2.21] for HTN). BMI had the weakest association with DM (2.46 [1.90–3.18]). Meanwhile, WHR was the least associated with HTN (1.59 [1.44–1.75]), which attenuated even further after adjustment for BMI (1.38 [1.24–1.53]). Residual confounding remained possible after accounting for several dietary patterns that might affect the outcomes. Based on the results, anthropometric indexes and the outcome biomarkers (HbA1c, SBP, and DBP) were weakly positively correlated, with all Spearman correlation coefficients of < 0.4, as shown in Supporting Table 1.

### 3.3. Comparison of Optimal Cutoffs, Sensitivity, and Specificity of Anthropometric Indexes With Current Standards

The width of AUC for each anthropometric index in diagnosing DM and HTN in adult Indonesians is presented in Table 4. For detecting DM, the most superior anthropometric index with the largest AUC is WC in women (AUC: 0.666 [0.624–0.707]) and WHtR in men (0.731 [0.679–0.784]). Meanwhile, for HTN, WHtR has the most superior diagnostic performance for both men (0.650 [0.629–0.671]) and women (0.615 [0.598–0.633]).

The upper part of Table 5 shows the optimal cutoffs for each anthropometric index along with their highest combination of sensitivity and specificity in detecting the presence of DM and HTN. The sensitivity and specificity values according to the current standard cutoffs are also shown in the lower part of the table for comparison.

For BMI, the current cutoffs show low sensitivity (45.8% and 32.8%) but high specificity (83.1% and 81.1%) for detecting DM and HTN, particularly in men. The lower proposed BMI cutoffs from the model (22.72 and 22.27 kg/m^2^) show higher sensitivity with lower specificity. A similar pattern was also observed for WC in men, where the proposed lower cutoffs led to improved sensitivity.

In comparison, the current WC cutoff for women produced high sensitivity (85.4% and 74.2%) but low specificity (37.3% and 37.3%) in diagnosing DM and HTN. Meanwhile, the new proposed cutoff balanced this discrepancy. Regarding WHR and WHtR, the current cutoffs produced good sensitivity to diagnose DM and HTN, particularly in women. The newly proposed cutoffs balance the performance by increasing the specificity. The measurement of anthropometric indexes using ROC and AUC in this study might have led to over- or underestimation, which was mitigated using the Youden index and cross-validation to determine the optimal cutoffs.

## 4. Discussion

In this nationwide study of the Indonesian population, the associations of anthropometric indexes with DM and HTN were investigated. Furthermore, the index with the best diagnostic performance in detecting the diseases in the population was identified. By using data from the large cohort of the IFLS, a comparison was made between the diagnostic performance of the current standard for obesity and the most optimal proposed cutoffs from the ROC model (Supporting Table 2).

The descriptive analysis showed that women had larger waist and hip circumferences than men, indicating the sex differences in body fat distribution [24]. Observation also showed prevalences of approximately 13% DM and 41% HTN, which were similar to results from several studies in Southeast Asia [25, 26]. According to the 2018 Indonesian Basic Health Research, the prevalence of HTN increased from 25.8% to 34.1% in 2013–2018. The prevalence of DM also slightly increased from 1.5% to 2.0% in the same period [27].

Compared to BMI, WC, and WHR, observation showed that WHtR had the strongest associations with both DM and HTN. This correlated with previous studies in developing nations, where WHtR had better discriminatory power than BMI, WC, and WHR in identifying DM, HTN, and MetS risks in adults [28–30], thereby serving as a screening tool for estimating visceral adiposity [19, 31–33]. Cutoffs of WhTR for predicting diseases in Malaysia, Singapore, and China were lower than in this study due to demographic situations and lifestyle factors. Similar to the Singaporean report [28], the discrepancies in AUC for several anthropometric indexes in this study were frequently minimal, accompanied by overlapping 95% CIs. However, the highest value was selected, as a greater AUC indicated superior performance. The superior diagnostic performance of WHtR in detecting DM and HTN could be partly explained by the strong association of WC with the amount of abdominal (visceral) fat, relative to an individual's height [34]. The accumulation of visceral fat in the abdomen and the subsequent high exposure of lipids to the liver have detrimental cardiometabolic consequences. This was because of the associations with glucose and lipid dysregulation, insulin resistance, and subclinical atherosclerosis, which served as precursors to DM and HTN [9]. Moreover, this study supported the widespread use of WHtR due to its adoption by the European Association for the Study of Obesity (EASO) as a diagnostic and staging criterion for obesity [35].

Regarding the optimal cutoffs for each anthropometric index, the results suggested that lower cutoffs for overall and abdominal obesity in the Indonesian population might be necessary. This was because DM and HTN were present at lower levels of BMI and WC than the current cutoffs. Using the universal, global cutoffs may be irrelevant in Indonesia because of the potential underestimation of cardiometabolic risks in the population, as shown by the low sensitivity. Therefore, lower cutoffs that were proposed might be crucial to achieving better sensitivity, as a good screening tool should have excellent sensitivity to detect the high risks of developing the disease of interest in many individuals [36]. The earlier development of cardiometabolic diseases at a lower amount of adipose tissue could be explained by the lipid overflow hypothesis. Specifically, the hypothesis proposes that Asian populations have a smaller subcutaneous fat compartment in the body, leading to earlier compartment exhaustion as obesity develops and the premature overflow of excess lipids to the visceral fat compartment [37]. This shows that detrimental effects of visceral fat appear at an earlier stage in the same amount of adiposity. Consequently, the proposed cutoff for Indonesia is distinctive and might be influenced by the different characteristics of the population from neighboring countries [28, 29, 38].

To decrease the prevalence of DM and HTN as national and global targets of Indonesia, this study recommends that WhTR be used as a screening tool along with BMI and WC due to the numerous benefits in the public health context [39]. Measurement of WhTR is more economical and practical because it requires solely a stadiometer.

Despite the significant contributions, this study has strengths and limitations. The strength is that the large and nationally representative sample enhances the robustness of the analysis. However, the cross-sectional and observational nature hinders the conclusion of any causality from the association model. The only available glucose parameter is HbA1c, which limits the diagnosis of DM to HbA1c criteria. Due to budget and logistical constraints, no fasting blood glucose or oral glucose tolerance test is performed on the participants, which are important parameters for diagnosis of DM [20]. These limitations may lead to an underestimation of DM prevalence in the population. Due to numerous missing data, the selection of subsamples may introduce selection bias that interferes with generalizability. Some self-reported questionnaires, particularly in dietary and lifestyle data, may elicit recall bias. In conclusion, this study showed that WHtR was the anthropometric index with the strongest associations with DM and HTN. WHtR also showed the best diagnostic performance for detecting these diseases in the Indonesian population. Moreover, nation-specific cutoffs with lower values for overall and abdominal obesity in Indonesia were recommended to estimate the cardiometabolic risks in the population accurately.

## Figures and Tables

**Table 1:** Population characteristics of the Indonesian family life survey.

Variables	Total (*n* = 7699)	Men (*n* = 3192)	Women (*n* = 4507)	*p*-value
Age (year)	52.59 ± 11.22 (35–94)	53.38 ± 11.51 (35–94)	52.03 ± 10.97 (35–89)	< 0.001
Living area (urban, %)	55.8	54.4	56.8	< 0.05
Marital status (married, %)	81.1	91.1	74.0	< 0.001
Level of education (high school or higher, %)	7.5	8.6	6.7	< 0.05
Working status (working, %)	77.2	89.8	68.3	< 0.001
Smoking status (smoker, %)	29.1	64.5	4.0	< 0.001
Physical activity (active, %)	18.4	18.4	5.1	< 0.001
Depression (yes, %)	19.7	18.1	20.9	< 0.05
Quality of sleep (poor, %)	24.2	21.9	25.8	< 0.05
Staple food consumption (days/week)	6.96 ± 0.44 (0–7)	6.97 ± 0.01 (0–7)	6.95 ± 0.01 (0–7)	0.124
Protein consumption (days/week)	2.29 ± 0.02 (0–7)	2.34 ± 0.02 (0–7)	2.24 ± 0.01 (0–7)	0.310
Sugary food consumption (days/week)	1.53 ± 0.03 (0–7)	1.45 ± 0.04 (0–7)	1.61 ± 0.03 (0–7)	0.260
Fruit consumption (days/week)	1.14 ± 0.01 (0–7)	1.09 ± 0.02 (0–7)	1.19 ± 0.01 (0–7)	0.520
Vegetable consumption (days/week)	2.41 ± 0.02 (0–7)	2.40 ± 0.03 (0–7)	2.42 ± 0.02 (0–7)	0.717
Height (cm)	154.05 ± 8.09 (124.60–185.40)	160.50 ± 6.31 (125–185)	149.48 ± 5.76 (124.60–174.50)	< 0.001
Weight (kg)	(56.66 ± 11.69) 22.00–104.90	58.30 ± 11.42 (22–104.90)	55.50 ± 11.73 (23.90–104.70)	< 0.001
Waist circumference (cm)	84.04 ± 11.63 (57–129.50)	81.92 ± 10.95 (57.00–129.50)	85.55 ± 11.86 (57–129.50)	< 0.001
Hip circumference (cm)	92.40 ± 9.75 (62.20–130.70)	89.35 ± 8.24 (63.30–130.70)	94.56 ± 10.15 (62.20–130.40)	< 0.001
Body mass index (kg/m^2^)	23.84 ± 4.46 (12.30–44.35)	22.55 ± 3.76 (13.40–42.94)	24.76 ± 4.69 (12.30–44.35)	< 0.001
Waist-to-hip ratio	0.91 ± 0.06 (0.58–1.39)	0.92 ± 0.06 (0.63–1.20)	0.90 ± 0.06 (0.58–1.39)	< 0.001
Waist-to-height ratio	0.55 ± 0.08 (0.34–0.87)	0.51 ± 0.07 (0.34–0.81)	0.57 ± 0.08 (0.38–0.87)	< 0.001
Systolic blood pressure (mmHg)	138.22 ± 24.25 (87.00–229)	137.08 ± 22.11 (91–229)	139.03 ± 25.64 (87.00–229.00)	0.075
Diastolic blood pressure (mmHg)	82.41 ± 12.34 (46.00–131.00)	81.87 ± 12.32 (50–131)	82.79 ± 12.35 (46.00–131.00)	< 0.001
Hypertension (%)	41.8	38.3	44.3	< 0.001
HbA1c (%)	5.81 ± 1.19 (3.50–14.80)	5.84 ± 1.13 (3.58–12.82)	5.79 ± 1.23 (3.50–14.80)	0.004
Diabetes mellitus (%)	13.2	13.4	13.0	0.844

*Note:* Values are presented as mean ± standard deviation (minimum–maximum) for continuous variables and proportion (%) for categorical variables; *p*-values were obtained from the Mann–Whitney *U* test for continuous variables and the chi-square test for categorical variables. These tests were performed to compare the characteristics between men and women.

**Table 2:** Population characteristics based on diabetes mellitus and hypertension status.

Variables	Mean ± SD		*p*-value	Mean ± SD		*p*-value
	HT (*n* = 3219)	Non-HT (*n* = 4480)		DM (*n* = 290)	Non-DM (*n* = 1913)	
Age (years)	56.36 ± 11.20	49.88 ± 10.42	< 0.001	58.76 ± 10.21	56.63 ± 11.77	0.004
Living area (urban, %)	42.8	57.2	0.046	15.3	84.7	0.001
Marital status (married, %)	39.1	60.9	< 0.001	13.3	86.7	0.852
Level of education (high school or higher, %)	36.7	63.3	0.011	17.3	82.7	0.143
Working status (working, %)	38.7	61.3	< 0.001	11.8	88.2	0.002
Smoking status (smoker, %)	35.8	64.2	< 0.001	11.1	88.9	0.080
Physical activity (inactive, %)	42.6	57.4	< 0.001	13.7	86.3	0.030
Depression (yes, %)	40.1	59.9	0.131	14.2	85.8	0.511
Quality of sleep (poor, %)	41.3	58.7	0.114	13.1	86.9	0.930
Staple food consumption (days/week)	6.96 ± 0.01	6.95 ± 0.02	0.652	6.93 ± 0.03	6.97 ± 0.01	0.188
Protein consumption (days/week)	2.21 ± 0.02	2.33 ± 0.01	0.270	2.25 ± 0.07	2.21 ± 0.03	0.675
Sugary food consumption (days/week)	1.40 ± 0.03	1.58 ± 0.04	0.320	1.44 ± 0.13	1.53 ± 0.05	0.509
Fruits consumption (days/week)	1.19 ± 0.02	1.13 ± 0.01	0.440	1.26 ± 0.07	1.25 ± 0.03	0.925
Vegetables consumption (days/week)	2.40 ± 0.03	2.43 ± 0.02	0.340	2.41 ± 0.10	2.33 ± 0.03	0.484
Weight (kg)	57.88 ± 12.32	55.78 ± 11.13	< 0.001	59.04 ± 11.85	53.98 ± 11.00	< 0.001
Height (cm)	152.96 ± 8.16	154.83 ± 7.95	< 0.001	153.16 ± 7.64	153.08 ± 8.22	0.888
BMI (kg/m^2^)	24.67 ± 4.62	23.24 ± 4.25	< 0.001	25.13 ± 4.51	23.00 ± 4.29	< 0.001
Waist circumference (cm)	86.57 ± 11.76	81.95 ± 11.09	< 0.001	90.18 ± 11.79	82.51 ± 11.35	< 0.001
Hip circumference (cm)	94.22 ± 9.89	90.89 ± 9.36	< 0.001	95.69 ± 9.17	91.13 ± 9.53	< 0.001
Waist-to-hip ratio	0.92 ± 0.06	0.90 ± 0.06	< 0.001	0.94 ± 0.07	0.90 ± 0.06	< 0.001
Waist-to-height ratio	0.57 ± 0.08	0.53 ± 0.08	< 0.001	0.59 ± 0.08	0.54 ± 0.08	< 0.001
HbA1c (%)	5.98 ± 1.32	5.67 ± 1.04	< 0.001	8.14 ± 1.66	5.46 ± 0.53	< 0.001
Systolic blood pressure (mmHg)	160.63 ± 20.03	122.12 ± 10.13	< 0.001	150.93 ± 27.11	140.20 ± 25.20	< 0.001
Diastolic blood pressure (mmHg)	92.21 ± 11.25	75.37 ± 7.25	< 0.001	85.46 ± 13.05	81.81 ± 12.37	< 0.001

*Note:* Values are presented as mean ± standard deviation and proportion (%); *p*-values were obtained from the Mann–Whitney *U* test for continuous variables and the chi-square test for categorical variables. These tests were performed to compare the characteristics of those with and without the diseases.

**Table 3:** Adjusted odds ratios of diabetes mellitus and hypertension in association with the anthropometric indices.

	**(a) Model 1. Odds ratios adjusted for sociodemographic and lifestyle factors**				
		**Body mass index**	**Waist circumference**	**Waist-to-hip ratio**	**Waist-to-height ratio**

Diabetes mellitus	Demographic, physical activity	2.462 (1.903; 3.184)^∗∗^	3.091 (2.360; 4.048)^∗∗^	2.514 (1.930; 3.276)^∗∗^	**3.166 (2.416; 4.150)** ^**∗∗**^
Hypertension	Demographic, smoking status, and physical activity	1.883 (1.710; 2.074)^∗∗^	1.911 (1.729; 2.113)^∗∗^	1.585 (1.436; 1.749)^∗∗^	**2.132 (1.928; 2.356)** ^**∗∗**^

	**(b) Model 2. Odds ratios adjusted for sociodemographic and lifestyle factors and BMI**				
		**Body mass index**	**Waist circumference**	**Waist-to-hip ratio**	**Waist-to-height ratio**

Diabetes mellitus	Demographic, physical activity, BMI		3.091 (2.360; 4.048)^∗∗^	2.005 (1.515; 2.654)^∗∗^	**3.166 (2.416; 4.150)** ^**∗∗**^
Hypertension	Demographic, smoking status, physical activity, BMI		1.616 (1.415; 1.847)^∗∗^	1.376 (1.240; 1.527)^∗∗^	**1.938 (1.703; 2.206)** ^**∗∗**^

*Note:* The anthropometric index with the highest significant OR was presented in bold (^∗∗^*p* < 0.001). Sociodemographic factors included age, marital status, education, working status, and living area.

**Table 4:** The area under the curve (AUC) of the anthropometric index (BMI, WC, WHR, WHtR) in determining DM and HTN in adults.

	BMI		Waist circumference		Waist-to-hip ratio		Waist-to-height ratio	
	Men	Women	Men	Women	Men	Women	Men	Women
Diabetes mellitus	0.678 (0.621–0.736)	0.629 (0.586–0.672)	0.715 (0.661–0.769)	**0.666 (0.624–0.707)**	0.699 (0.645–0.754)	0.639 (0.595–0.683)	**0.731 (0.679–0.784)**	0.661 (0.619–0.702)
Hypertension	0.614 (0.593–0.636)	0.565 (0.547–0.583)	0.636 (0.615–0.657)	0.594 (0.576–0.612)	0.604 (0.583–0.626)	0.576 (0.558–0.594)	**0.650 (0.629–0.671)**	**0.615 (0.598–0.633)**

*Note:* The anthropometric index with the highest AUC value was presented in bold for men and women.

**Table 5:** Cutoff points, sensitivity, and specificity for BMI, WC, WHR, and WHtR predictive of DM and HTN.

**(a) Optimal cutoff points for BMI, WC, WHR, and WHtR predictive of DM and HTN**																								
	**BMI**						**WC**						**WHR**						**WHtR**					
	**Cutoff (kg/** **m** ^2^ **)**		**Se (%)**		**Sp (%)**		**Cutoff (kg/** **m** ^2^ **)**		**Se (%)**		**Sp (%)**		**Cutoff (kg/** **m** ^2^ **)**		**Se (%)**		**Sp (%)**		**Cutoff (kg/** **m** ^2^ **)**		**Se (%)**		**Sp (%)**	
	**Men**	**Women**	**Men**	**Women**	**Men**	**Women**	**Men**	**Women**	**Men**	**Women**	**Men**	**Women**	**Men**	**Women**	**Men**	**Women**	**Men**	**Women**	**Men**	**Women**	**Men**	**Women**	**Men**	**Women**

Diabetes mellitus	22.72	24.85	64.30	61.00	64.00	60.50	83.35	87.45	66.10	63.40	66.00	62.90	0.93	0.92	65.20	61.00	64.40	60.50	0.52	0.59	65.70	63.40	65.60	63.50
Hypertension	22.27	24.54	59.30	54.40	59.20	54.40	81.25	85.95	59.80	56.30	60.30	56.80	0.92	0.91	57.60	55.00	57.60	55.00	0.51	0.57	61.20	58.30	61.30	58.30

**(b) Sensitivity and specificity for BMI, WC, WHR and WHtR predictive of DM and HTN based on the standard cutoff points** ^**∗**^																								
	**Cutoff (kg/** **m** ^2^ **)**		**Se (%)**		**Sp (%)**		**Cutoff (kg/** **m** ^2^ **)**		**Se (%)**		**Sp (%)**		**Cutoff (kg/** **m** ^2^ **)**		**Se (%)**		**Sp (%)**		**Cutoff (kg/** **m** ^2^ **)**		**Se (%)**		**Sp (%)**	
	**Men**	**Women**	**Men**	**Women**	**Men**	**Women**	**Men**	**Women**	**Men**	**Women**	**Men**	**Women**	**Men**	**Women**	**Men**	**Women**	**Men**	**Women**	**Men**	**Women**	**Men**	**Women**	**Men**	**Women**

Diabetes mellitus	25	25	45.8	58.10	83.1	61.50	90	80	45.40	85.40	83.20	37.30	0.9	0.85	75.00	92.70	45.90	20.10	0.5	0.5	76.80	92.10	53.80	19.70
Hypertension	25	25	32.80	52.20	81.10	58.30	90	80	32.40	74.20	83.90	37.30	0.9	0.85	67.60	85.20	46.90	23.60	0.5	0.5	64.70	87.90	56.40	23.10

*Note:* Se, sensitivity; Sp, specificity; WHtR, waist-to-hip ratio; HTN, hypertension.

Abbreviations: BMI = body mass index, DM = diabetes mellitus, WC = waist circumference, WHR = waist-to-hip ratio.

^∗^We used the Asian population-specific cutoffs of 25 kg/m^2^ (for BMI); 80 cm in women and 90 cm in men (for waist circumference); 0.9 in men and 0.85 in women (for WHR); and 0.5 for WHtR.

## Data Availability

The data that support the results are openly available in IFLS at https://www.rand.org/well-being/social-and-behavioral-policy/data/FLS/IFLS/download.html.

## Supporting Information

Supporting Information 1 Supporting Table 1: Spearman correlation coefficients of the relationship between anthropometric indexes and the marker of DM and HTN.

Supporting Information 2 Supporting Table 2: Receiving operating characteristics (ROC) curve analysis for detecting DM and HTN.
