# Supplementary material for: Redefining Obesity in the Indonesian Population: The Critical Role of Waist-to-Height Ratio in Screening for Diabetes Mellitus and Hypertension
Source: J Nutr Metab. 2025 Sep 2;2025:5815261. doi: 10.1155/jnme/5815261 (PMC12419920; doi:10.1155/jnme/5815261)
Supplement: Supporting Information 1 — Supporting Table 1: Spearman correlation coefficients of the relationship between anthropometric indexes and the marker of DM and HTN. [file 5815261.f1.docx]

**Supplemental Table 1. Spearman Correlation Coefficients of the Relationship between Anthropometric Indices and the Marker of DM and Hypertension**

|  | **BMI (kg/m^2^)** | **WC (cm)** | **WHR** | **WHtR** |
| --- | --- | --- | --- | --- |
| **Total Population (n=7699)** |  |  |  |  |
| HbA1C (%) | 0.194 | **0.246** | 0.223 | 0.213 |
| Systolic Blood Pressure (mm Hg) | 0.161 | 0.220 | 0.186 | **0.253** |
| Diastolic Blood Pressure (mm Hg) | **0.297** | 0.293 | 0.186 | 0.295 |
| **Women (n=4507)** |  |  |  |  |
| HbA1C (%) | 0.204 | **0.257** | 0.189 | 0.240 |
| Systolic Blood Pressure (mm Hg) | 0.121 | 0.185 | 0.175 | **0.232** |
| Diastolic Blood Pressure (mm Hg) | 0.269 | 0.257 | 0.145 | **0.276** |
| **Men (n=3192)** |  |  |  |  |
| HbA1C (%) | 0.223 | 0.266 | 0.274 | **0.281** |
| Systolic Blood Pressure (mm Hg) | 0.234 | 0.263 | 0.218 | **0.297** |
| Diastolic Blood Pressure (mm Hg) | **0.337** | 0.333 | 0.257 | 0.332 |

All are significant at the level of <0.001 (2-tailed); the anthropometric index with the highest correlation coefficient for each relationship was presented in **bold**; BMI Body Mass Index; WC Waist Circumference; WHR Waist-Hip Ratio; WHtR Waist-Height Ratio.
