# Supplementary material for: Redefining Obesity in the Indonesian Population: The Critical Role of Waist-to-Height Ratio in Screening for Diabetes Mellitus and Hypertension
Source: J Nutr Metab. 2025 Sep 2;2025:5815261. doi: 10.1155/jnme/5815261 (PMC12419920; doi:10.1155/jnme/5815261)
Supplement: Supporting Information 2 — Supporting Table 2: Receiving operating characteristics (ROC) curve analysis for detecting DM and HTN. [file 5815261.f2.docx]

**Supplemental Table 2. The Receiving Operating Characteristics (ROC) curve analysis for detecting DM and HTN**


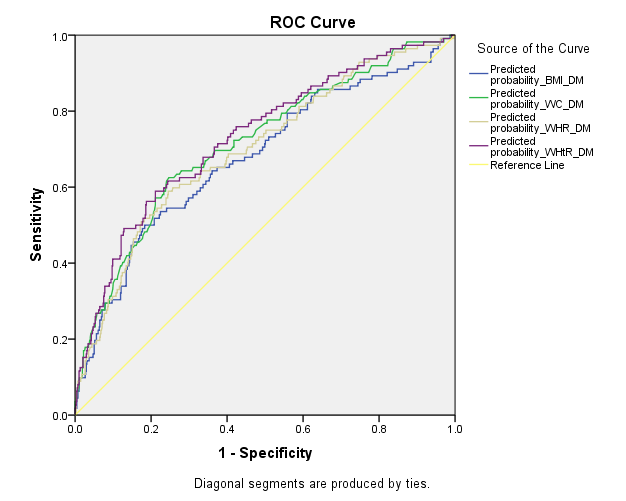


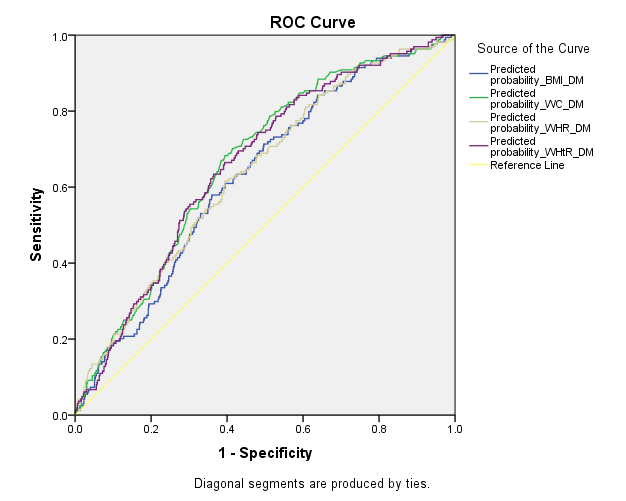


**Figure 1a. ROC Curve for Diabetes Mellitus in Men**

**Figure 1b. ROC Curve for Diabetes Mellitus in**

**Women**


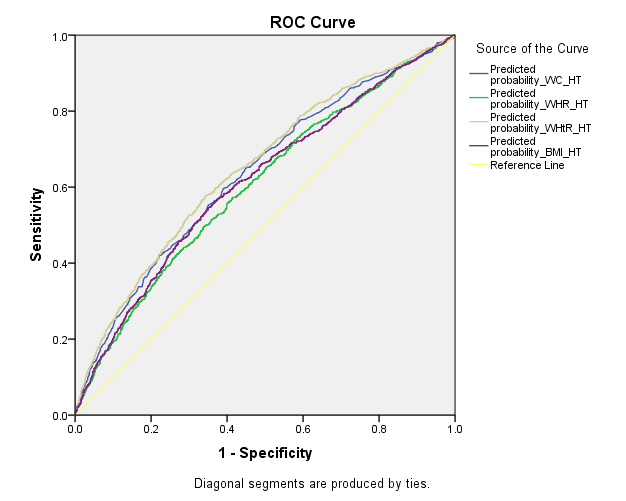


**Figure 2a. ROC Curve for Hypertension in Men**


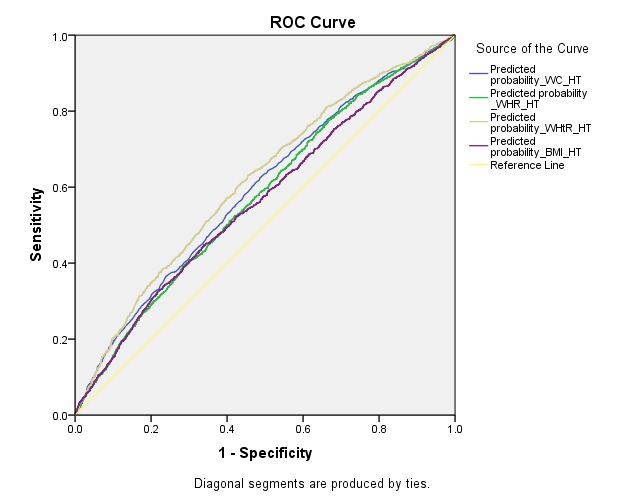


**Figure 2b. ROC Curve for Hypertension in Women**
